# Urban Food Takeaway Vitality: A New Technique to Assess Urban Vitality

**DOI:** 10.3390/ijerph18073578

**Published:** 2021-03-30

**Authors:** Bahram Zikirya, Xiong He, Ming Li, Chunshan Zhou

**Affiliations:** 1 College of Tourism, Xinjiang University, Urumqi 830049, China; zikeliya5@mail2.sysu.edu.cn; 2 School of Geography and Planning, Sun Yat-sen University, Guangzhou 510275, China; hexiong6@mail2.sysu.edu.cn (X.H.); liming57@mail2.sysu.edu.cn (M.L.); 3 Key Laboratory of the Sustainable Development of Xinjiang’s Historical and Cultural Tourism, Xinjiang University, Urumqi 830046, China

**Keywords:** urban spatial structure, quantitative analysis, spatial correlation, catering industry, multi-source data

## Abstract

As one of the most important criteria for measuring the quality of urban life and the environment, urban vitality has become the focus of urban-related research and related disciplines with an increasing number of advocates for the rapid and harmonious development of urban cities. Urban takeaway can represent urban vitality, but studies have not investigated this in a quantitative manner. Furthermore, current studies rarely focus on or even mention the urban food takeaway vitality generated by the spatial distribution of urban takeaway. This study first calculated the vitality of urban takeaways based on the urban takeaway distribution, building footprint, Open Street Map (OSM) data, and the Rapidly Exploring Random Tree (RRT). Then, the urban vitality was obtained using Tencent-Yichuxing data and night-time light data, followed by a spatial correlation analysis between the urban takeaway vitality and urban vitality. Finally, the results for Beijing, Shanghai, and Guangzhou were compared, and the following conclusions were drawn: (1) there is a significant spatial correlation between the urban takeaway vitality and urban vitality, but the correlation varies in different cities at different times; and (2) even in the same city, different road and building densities have an impact on the correlation. The urban takeaway vitality proposed in this study can be used as a new index to evaluate the urban vitality, which has important theoretical and practical significance for the sustainable development of future urban cities.

## 1. Introduction

Although continuous urban expansion renders urban boundaries increasingly larger, there has been a decrease in the density of some cities [1]. This strange phenomenon has caused scholars to reflect on rapid urbanization. In the past 30 years, China’s urban development model, based on land finance, has realized significant achievements in urban construction in a relatively short period [2]. At present, urban planning in China has transformed from incremental planning to stock planning, such that urban renewal, urban protection, and revitalization are gradually attracting attention [3,4], What’s more, the harmonious and rapid development of urban cities has also been one of the most important topics of discussion. Due to the fact that urban system is a huge urban life entity, the better it is, the better the living environment it can provide. Therefore, previous studies widely accept that the vitality of an urban system is one of the important standards to measure the quality of urban life [5]. The vitality of the city is closely related to the spatial quality of the city, which is reflected in the fact that complex land use degrees, high-density construction environments, and appropriate space environmental facilities are used to improve the sustainable attraction of a city to its surrounding area, thus enhancing the urban vitality [6,7].

Urban vitality is not a new concept; it has been mentioned in urban planning and construction studies for a long time [8]. Both Gehl and Jacobs hold the idea that urban vitality comes from the activities of people and population in the urban space [9,10]. As the most original energy within a city, urban vitality can not only directly represent the population activity at different times in different cities [11], but it can also indicate the basic representation of the quality of urban life as well as the spatial quality situation owing to its derivation from urban morphology, urban function, and urban activity [12,13]. In the context of the existing research on urban vitality, urban vitality can be essentially defined as the activity intensity of the urban population in the urban space. The denser the population activities in urban space, the more dynamic the city is [13,14,15]. Urban vitality has become a critical concern for governors and planners in terms of rationally quantifying this concept, which can play an essential role in achieving updates to the sustainable development in both theoretical and practical manners in urban cities [16,17]. Along with the land use, urban landscapes, including urban buildings and environmental infrastructure, are also considered as important data sources for evaluations of urban vitality [18,19]. However, rather than only accounting for the urban landscape when analyzing urban vitality, more attention should be focused on individual living in urban cities [20], which is resulting from the fact that population activity is the most direct feedback to the spatial quality and vitality of the city.

With the extensive application of open source big data in relevant studies on urban cities and geography, data that can represent population mobility and interaction, including Point of Interest (POI) data [21], Location Based Services (LBS) data [22], mobile signalling data [23], transportation card data [24], Global Positioning System (GPS) data [25], and social media punch dates [26], all provide reliable sources for the quantitative analysis of urban vitality [27]. The merits and demerits of these data are mainly embodied in the following expects: POI data, as a geographic virtual representation of an urban entity, are of significant geographic reliability, but are more inclined to represent spatial forms [28]; for mobile signalling data, although a large number of locator data can be obtained in a relatively shorter period using a signal station, mobile signalling data still have certain shortcomings, such as lower reliability and inaccurate positioning information [29]; for GPS data, the positioning information obtained by GPS has a high accuracy and interference immunity, but its elevated operating costs make its use inappropriate for refined studies in small-scale regions [30]; as for social media punch data, using these types of data to represent the spatial activity of the elderly and children is rather difficult due to significant age differences, despite a relatively higher number of sample data and better accessibility to it [31]; for transportation card data, this type of data is restricted to the areas among transport lines, although it can represent living spaces and population movement [32]; and finally, as one of the LBS data types that derives from the Amap positioning service, Tencent-Yichucing data, can always generate well-positioned movement data for population positions, as long as the apps (including instant messaging software, such as WeChat and QQ) that belong to Tencent are used. This sample data has a wider coverage and higher accuracy, which can be used to represent the daytime vitality of an urban city [33,34].

There is a significant difference in the urban vitality at various time frames in urban areas, especially between the day and night [35]. The relatively more active mobility and population interactions allow for easier calculations of the daytime utility value while, in contrast, reduced population mobility at night renders it more difficult to accurately calculate the night vitality value [36]. As a type of satellite remote-sensing data, night-time light data is considered not only the most prominent representation of urban night vitality, but also one of the most prominent indicators that represents urban social economic activities. Therefore, night-time light data is widely used to examine the human activity intensity and urban spatial variation [37,38,39,40]. Previous studies have also shown that there are significant correlations among the night-time light value, economic manufacturing, and energy consumption, as well as open activities [41,42,43]. For the Defence Meteorological Program/Operational Line-Scan System (DMSP/OLS) and Suomi National Polar-orbiting Partnership/Visible Infrared Imaging Radiometer Suite (NPP/VIIRS), the lower spatial resolution of these data result in lower degree of fineness in meso- and micro-scale regions [44,45]. The spatial resolution of the newly released Luojia-01 data is as high as 130 m, with significant improvements to the fineness [46]. Therefore, Tencent-Yichuxing data and night-time light data are respectively widely used to represent DV and NV in urban settings.

As a type of small catering service that first emerged in the United States in the 1990s, takeaway service has been widely embraced by the populace due to its convenient service mode, cheap consumption price, and rapid order pattern [47]. Moreover, takeaway service is becoming an integral part of people’s urban life with urban development, followed by substantial living pressure. More and more people are using takeaway services to meet their daily food needs in order to save time for work and rest. This kind of food consumption behavior has become an important part of urban life, especially in China which is rapidly urbanizing. What’s more, the addition of a large number of online takeaway platforms makes urban takeaway gradually become an indispensable part of modern urban life [48]. In 2020, the total transaction volume of China’s takeaway industry has exceeded 650 billion yuan, which further indicates the important position of takeout in the urban catering industry [49]. However, at present, studies on urban takeaway either analyze this business behavior from an economic perspective [50], or consider how to optimize takeaway services from a food consumption safety perspective [51]. Few studies explore the relationship between takeaway services and urban cities from a geographical space perspective, not to mention the lack of studies on the development of spatial quality reflected by takeaway business. Takeaway is not only a way of life, but also a form of expression that reflects the urban population and the development of spatial quality. Food consumption, including urban takeaway, is an indispensable necessity for people in the city. As a consumption activity of urban population in the city, urban takeaway will be one of the important sources of urban vitality [13].

From an urban geographic space perspective, the connection between the takeaway service and urban space is mostly limited to qualitative analyses [52]. Although the spatial correlation between the takeaway service and urban space has been explored in several studies, previous studies have not carried out in-depth analyses of the strength of such correlations and their internal factors [53,54]. With the expansion of this type of research, relevant studies on urban takeaway have begun to incorporate quantitative factors, but the research still focuses on delivery route selection and takeaway optimization [55,56]. Although the core of takeaway delivery is in the ‘service area,’ previous studies have not discussed the relationship between different delivery service areas and urban space. Enhancing the vitality of a city lies in continuously attracting the surrounding population and its resources [57,58]. As urban takeaway develops alongside cities, takeaway tends to develop well in densely populated areas [59]. Like urban vitality, different materials and spatial forms in urban space have their own scope of vitality [60], such as block vitality [61], economic vitality [62], and spatial vitality [63], etc. Similarly, the special catering behavior of takeaway should also have its own range of vitality. Analogous to the definition of urban vitality. The vitality of urban takeaway can be defined as the sum of the number of takeaway businesses that a certain city unit can accept within a certain time. Then, the vitality of urban takeaway and urban vitality both belong to the urban space, is there a certain spatial connection? if so, does the takeaway vitality itself correlate with the vitality of the city?

This study discusses two main questions: (1) how can we quantify urban takeaway and calculate the distribution of the vitality value for urban takeaway? and (2) what is the spatial correlation between the vitality of urban takeaway and the vitality of cities at different times (day and night)? To obtain answers to these questions, this study uses urban takeaway distribution data, road data, and building footprint data to quantitatively analyze the urban takeaway distribution range with the Rapidly Exploring Random Tree (RRT) algorithm to quantify the vitality value of takeaway. The daytime and night-time vitality of urban areas are then calculated using population mobility data and night-time light data. Next, the local spatial autocorrelation is used to analyze the local relationship between the urban takeaway vitality and urban vitality at different times, with a final analysis of the local relationships. This study uses Beijing, Shanghai, and Guangzhou, which are the fastest growing cities in China, as examples to analyze and summarize the urban takeaway and urban vitalities, which can provide a reference for the harmonious and rapid development of other cities.

## 2. Materials and Methods

### 2.1. Study Area and Data Source

**Figure 1:**
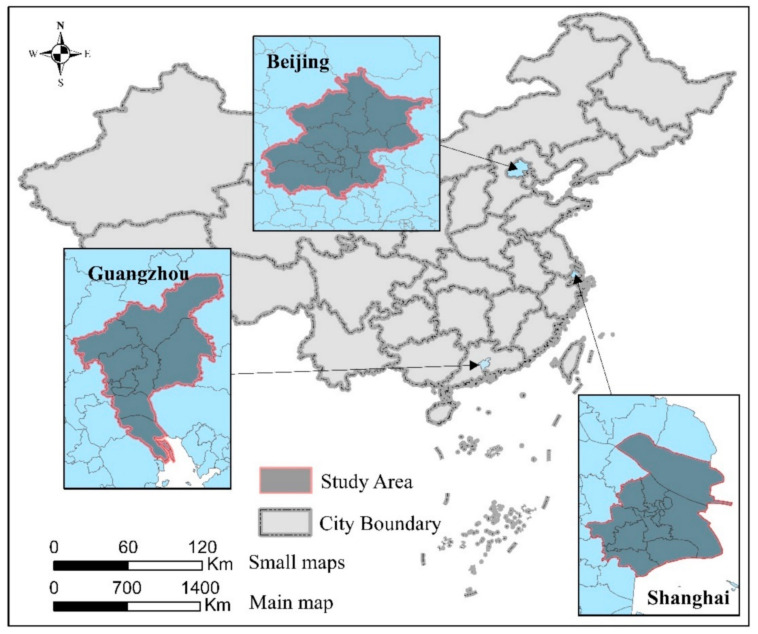
Study Area.

This study analyzed Beijing, Shanghai, and Guangzhou (Figure 1), which rank among the top three strongest cities based on comprehensive strength in China [56]. Large-scale urban agglomerations, such as the Beijing-Tianjin-Hebei metropolitan area, Yangtze River Delta economic belt, and Pearl River Delta economic belt have formed, owing to the solid economic foundation and considerable political resources of these cities. As the most developed cities on the Chinese mainland, the spatial distributions of Beijing, Shanghai, and Guangzhou are critical [57]. In addition, as the first-tier metropolis in China, Beijing, Shanghai and Guangzhou have the most obvious level of internationalization [64]. Taking the most international metropolis in China as the case points will also help to promote and apply the study results. What’s more, the case study of these three cities can significantly contribute to the comprehensive understanding of the spatial relationship between the vitality of the takeaway industry and the urban vitality, as well as contribute to discussions on whether such a spatial relationship is universal [65].

Available open source data used in this study mainly included distribution data for takeaway businesses in Beijing, Shanghai, and Guangzhou; OSM data; building footprint data; night-time light data; and Tencent-Yichuxing data. In terms of the distribution data for takeaway businesses, as of December 2020, the number of takeaway businesses registered on Meituan in Beijing, Shanghai, and Guangzhou was 18,159, 15,009, and 14,638, respectively (https://www.meituan.com/, accessed on 31 December 2020). OSM data mainly refer to road information for the three cities in 2020, including detailed road information on the highway classes, such as arterial highway, secondary highway, tertiary highway, national trunk highway, provincial trunk highway, county road, and township road. Building footprint data were mainly derived from Amap (www.amap.com, accessed on 31 December 2020). By accessing the Amap Application Programming Interface (API), we obtained the building space coordinates, basic outlines, and floor details for the infrastructure in Beijing, Shanghai, and Guangzhou in 2020. Night-time light data for Beijing, Shanghai, and Guangzhou, derived from LuoJia-01 data with a spatial resolution of 130 m from October 2018 to October 2020, were obtained from the Hubei Data and Application Network of the High-Resolution Earth Observation System (http://59.175.109.173:8888/index.html, accessed on 31 December 2020), with a processed monthly average. Population mobility data: population is not only one of the most important components of a city, but also the main carrier of social culture. Population mobility is complex and multilateral. In order to better describe the characteristics and structure of population mobility, based on the reference of Disparity Filter Algorithm [66], this manuscript conducts clustering and differentiation feature extraction for the population mobility data in the research area, so as to carry out the comparative study in different areas. It can be seen from the screening results that the proportion of young and middle-aged people who using urban takeaway service is the largest. Besides, the population mobility data comes from Tencent-Yichuxing data. Tencent-Yichuxing data, which are population movement data with a spatial resolution of 25 m × 25 m based on Tencent APP’s analysis of a user’s location, were provided by Tencent’s positioning big data service window (http://heat.qq.com/index.php, accessed on 31 December 2020). Table 1 lists a statistical description of all the data.

### 2.2. Methods

**Figure 2:**
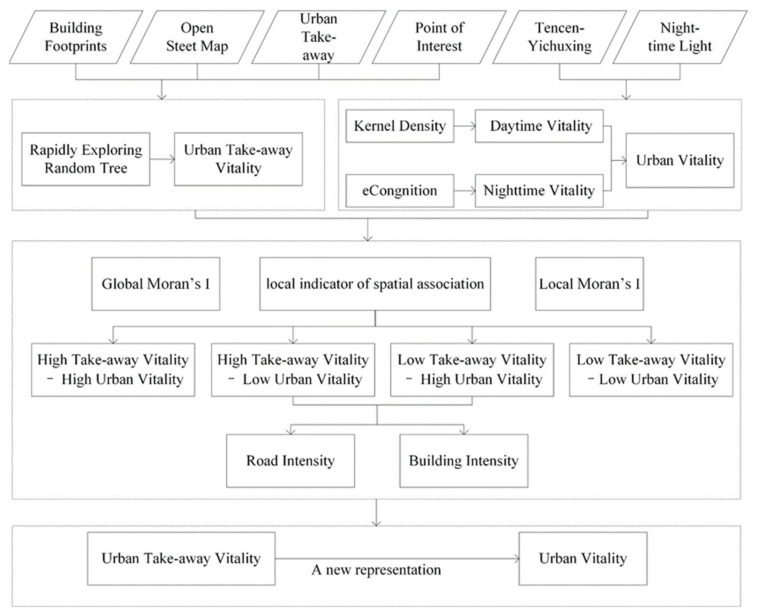
Study Framework.

The spatial vitality of urban takeaway businesses is defined by the relevant spatial relationship between the vitality of a takeaway business and the urban vitality, which is analysed based on the quantitative method shown in Figure 2. This study was divided into three part: first, the urban daytime vitality and night-time vitality were calculated using the Tencen-Yichuxing data and LuoJia-01 data; second, the RRT was used to quantitatively calculate the takeaway vitality by combining the OSM and building footprint data; and finally, the spatial correlation analysis between them was analysed. In this study, the definition of the takeaway vitality value is the number of takeaway orders that can be placed online in any physical location in a city. Therefore, the takeaway vitality value directly depends on the delivery time and order distance.

#### 2.2.1. Daytime Vitality Quantification

As one of the data types that can best represent the flow and change in population over a short period, Tencen-Yichuxing data can represent the urban DV. In addition, kernel density analysis was used to calculate the agglomeration of point and line elements in space to simulate the continuous changes in elements in space [67], which has a wide range of applications in urban spatial distribution analysis. The final density value, $p_{i}$, was calculated as follows:

(1) $$p_{i}=\frac{1}{n\pi R^{2}}\times \overset{n}{\underset{j=1}{\sum }}k_{j}{\left(1-\frac{D_{ij}^{2}}{R^{2}}\right)}^{2}$$

In nuclear density analysis, different bandwidths have a significant influence on the results of the analysis. A larger kernel density bandwidth can reflect the macroscopic spatial structure of the region while a smaller bandwidth can identify the agglomeration in the microscopic region. Therefore, to better determine the bandwidth of the kernel density analysis, Silverman’s rule of thumb was used in this study to calculate the bandwidth of the kernel density analysis:

(2) $$R=0.9\times min(SD,\;D_{m}\times \sqrt{\frac{1}{ln2}})\times n^{-0.2}$$

where n is the number of element points, $D_{m}$ is the median of the distance from each element point and the average centre, and SD is the standard distance of the element points. Finally, the average value obtained by weighting the peak value and minimum value of the population mobility from the Tencent-Yichuxing data in different periods was set as the DV value.

#### 2.2.2. Night Vitality Quantification

The night-time light data use the brightness value (digital number-DN) of night-time city lights to indicate the strength of night-time city activities. Compared with DMSP/OLS and NPP/VIIRS, Luojia-01 data has a more convenient data acquisition method and more accurate spatial resolution, which can better represent the spatial pattern of cities at night. Luojia-01 data were resampled to maintain the same spatial resolution as the Tencent-Yichuxing data; therefore, the average value of the lighting from October 2018 to October 2020 was set as the NV value.

#### 2.2.3. RRT Algorithm

As a single query motion planning algorithm based on random sampling, the RRT algorithm has a wide range of applications in robot motion [68,69]. RRT roots at a configuration, $q_{init},$ randomly samples the state node, $q_{rand}$, in the space state, C, passes all of the nodes of the Tree, and finds the node, $q_{near}$, closest to $q_{rand}$. Then, the robot is driven to transfer a fixed step from the direction of $q_{near}$ to the node, $q_{new}$. If a collision with an obstacle occurs during the transfer process, the node, $q_{new}$, and the corresponding edge are added to the Tree. This process is repeated until the goal state, $q_{goal}$, is reached; finally, the goal state, $q_{goal}$, is traced back to the root node, $q_{init}$, to obtain the final planned path. The core steps of the search process are as follows:

(3) $$q_{rand}\to random\_sample\left(\;\right)$$

(4) $$q_{near}\to near\_neighbor\left(q_{rand},T\right)$$

(5) $$q_{new}\to sTree\left(q_{near},q_{rand}\right)$$

The takeaway vitality value calculated in this study depends on the takeaway delivery time and delivery distance. The Meituan APP shows that an order exceeds the deliverable distance when the delivery time exceeds 45 min. Therefore, the takeaway vitality value is all of the locations that a takeaway merchant can reach within 45 min.

**Figure 3:**
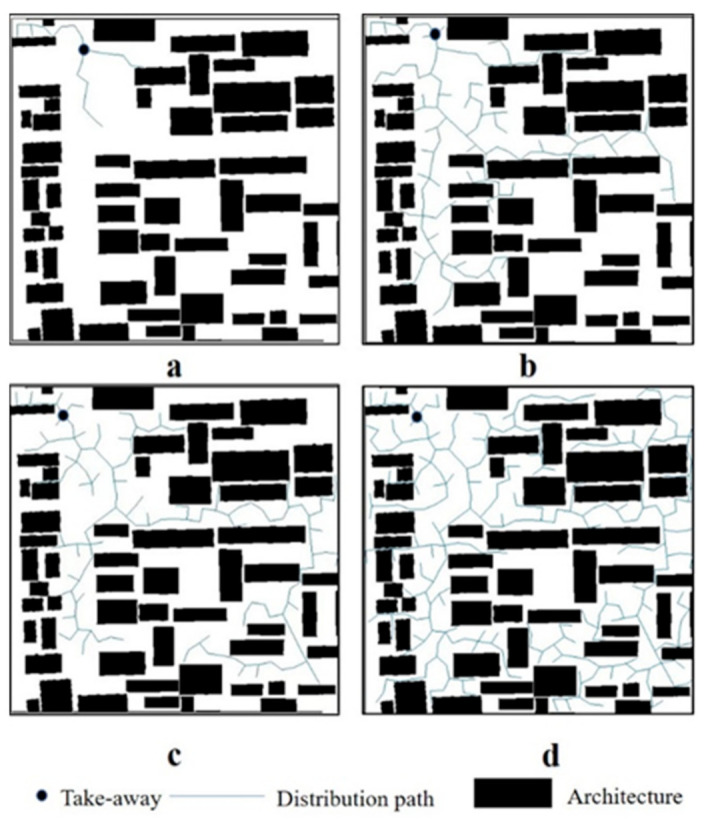
Regional planning model for takeaway delivery (From **a**–**d**, takeaway delivery area gradually increases).

Assuming that the delivery speed of the takeaway is equal, after the introduction of the RRT algorithm, $q_{init}$ is the location of the takeaway merchant, $q_{rand}$ is the pre-delivery sampling location,$\;q_{near}$ is the newly emerging delivery location, urban buildings are the obstacles that may exhibit collision during the transfer process, and $q_{new}$ is the delivery location that appears after collision. Figure 3 shows the delivery distance path planning model.

#### 2.2.4. Local Indicator of Spatial Association

As one of the important methods for evaluating local spatial autocorrelation [70,71], the local indicator of spatial association (LISA) can detect clustering outliers in geographic space to determine whether there is spatial autocorrelation between two entities [72]. Using LISA to evaluate the local interaction between the vitality of takeaway delivery and urban vitality can identify the high and low value distributions of vitality and the degree of correlation between the vitality of takeaway delivery and urban vitality in a geographic space. LISA is divided into univariate and bivariate. The univariate LISA can be represented as follows:

(6) $$I_{i}=\frac{Z_{i}}{S^{2}}\overset{n}{\underset{j\neq 1}{\sum }}w_{ij}Z_{j}$$

(7) $$Z_{i}=X_{i}-\bar{X},\;Z_{j}=X_{j}-\bar{X},\;\;S^{2}=\frac{\sum {\left(X_{i}-\bar{X}\right)}^{2}}{\sigma }$$

where $w_{ij}$ is the spatial weight value of units i and j, n is the sum of all areas in the study area, $I_{i}$ is the local Moran’s I index of the i-th area, $X_{i}$ is the value of unit i, $\bar{X}$ is the average value, and σ is the standard deviation. When the values of $Z_{i},\overset{n}{\underset{j\neq 1}{\sum }}w_{ij}Z_{j}$, and $I_{i}$ are greater than 0 or less than 0, Moran’s I index scatter plots located in the fourth quadrant are generated. This local autocorrelation is only applicable to univariate. If there is a bivariate in the geographic space, a bivariate LISA is required, which can be expressed as follows:

(8) $$I_{k,l}^{i}=Z_{i},k\overset{n}{\underset{j=1}{\sum }}w_{ij}\times Z_{j,l}$$

(9) $$Z_{i,k}=\frac{\left(X_{i,k}-{\bar{X}}_{k}\right)}{\sigma k},Z_{i,l}=\frac{\left(X_{i,l}-{\bar{X}}_{l}\right)}{\sigma l}$$

where k and l are variables. According to the statistics of the bivariate LISA, the Moran’s I scatter plot with four quadrants and high- and low-density clustering mapping related to the vitality can be obtained, including areas with high and high vitality (H-H), high and low vitality (H-L), low and high vitality (L-H), and low and low vitality (L-L).

## 3. Results

### 3.1. Urban Vitality

**Figure 4:**
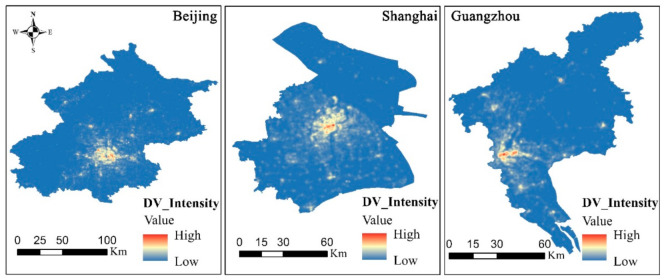
Daytime vitality distribution in Beijing, Shanghai, and Guangzhou.

Figure 4 shows the DVs of Beijing, Shanghai, and Guangzhou, which were obtained using the monthly average Tencent-Yichuxing data in December 2020. Based on Figure 4, Beijing’s DV is mainly concentrated within the fifth ring road while most places of interest, such as the Forbidden City, are mainly concentrated within first ring road, with reduced population mobility, except for tourists. The greatest population mobility is mainly concentrated within the second and third ring roads. Therefore, the high DV value is distributed around the second ring road to the third ring road. Shanghai’s DV is mainly distributed in the Jing’an, Huangpu, and Putuo districts, among others. High DV values are mainly concentrated in the Jing’an District. The DV in Guangzhou is mainly distributed in the Haizhu, Baiyun, and Yuexiu districts, where the Haizhu District is a high DV concentration area. Based on the distribution regions of the DV values, Shanghai has the most extensive DV distribution region, followed by Guangzhou and finally Beijing. In terms of the high DV value agglomeration, Guangzhou has the most notable DV value agglomeration, followed by Shanghai and finally Beijing. Therefore, in the daytime, Shanghai has the greatest population mobility, but it is relatively dispersed; the population mobility area of Guangzhou is relatively dense, whereas the urban DV of Beijing is the lowest.

**Figure 5:**
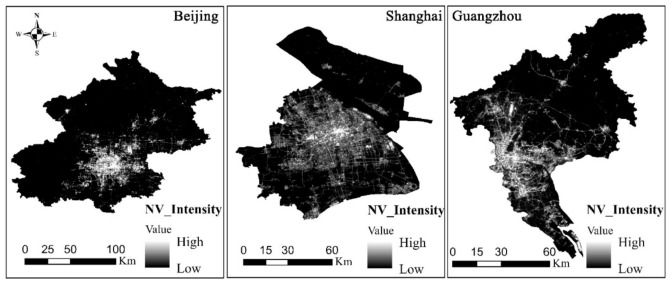
Night vitality distribution in Beijing, Shanghai, and Guangzhou.

Figure 5 shows the NV distribution of Beijing, Shanghai, and Guangzhou, which was obtained using the monthly mean LuoJia-01 night-time light data from December 2018 to December 2020. Based on Figure 5, for the distribution of high and low NV values, Beijing has the highest NV value, followed by Shanghai and finally Guangzhou. From the perspective of the distribution range for night-time light, Shanghai has the most extensive distribution range, followed by Guangzhou and finally Beijing. We note that there are certain areas in both Beijing and Guangzhou that have no NV values. The main reason for this is that compared with Shanghai, Beijing and Guangzhou include a large number of suburbs except for a few core areas, where the night vitality is further reduced at night, making the NV value lower. While except for Chongming Island, the territorial jurisdiction in Shanghai all belongs to downtown area due to its special geographical environment.

### 3.2. Takeaway Business Vitality

In the takeaway delivery model shown in Figure 3, the takeaway vitality value refers to all of the locations that the takeaway merchant can reach within 45 min. In cities, takeaway delivery depends on the density of urban buildings and road conditions. Theoretically, the more accessible that the roads in a city are from all directions, takeaway vehicles can achieve greater delivery distances under the assumption of a moderate building density. The number of takeaway restaurants in Beijing, Shanghai, and Guangzhou is 18,159, 14,638, and 15,009. In terms of the road conditions, Shanghai has the best road coverage while Beijing and Guangzhou have relatively imperfect road coverage. From a building footprint perspective, the total number of buildings in Beijing is 578,453, 613,369 in Shanghai, and at least 473,761 in Guangzhou.

**Figure 6:**
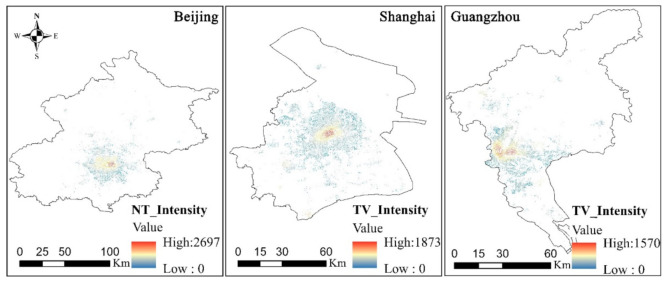
Distribution of the urban takeaway vitality in Beijing, Shanghai, and Guangzhou.

By introducing the takeaway restaurants and the roads and buildings in Beijing, Shanghai, and Guangzhou into the RRT model, the takeaway vitality value distribution map of each region during delivery periods can be obtained, as shown in Figure 6. Based on the distributions of the vitality values in Beijing, Shanghai, and Guangzhou in Figure 6, the highest takeaway vitality value is 2697 in Beijing, 1873 in Shanghai, and 1570 in Guangzhou. From the perspective of the range distribution for the takeaway vitality value, the takeaway vitality value in Shanghai is distributed in all regions, except for Chongming Island, while a few regions in Beijing and Guangzhou have no takeaway vitality value distribution. In terms of the value of the takeaway vitality, Beijing has the highest takeaway vitality while Shanghai has the widest distribution. Referring to the road and building footprint data, the main reason for the concentrated takeaway vitality in Beijing and Guangzhou is that the roads restrict the takeaway distribution, which yields relatively lower vitality values.

### 3.3. Spatial Relationship between Takeaway Vitality and Urban Vitality

**Figure 7:**
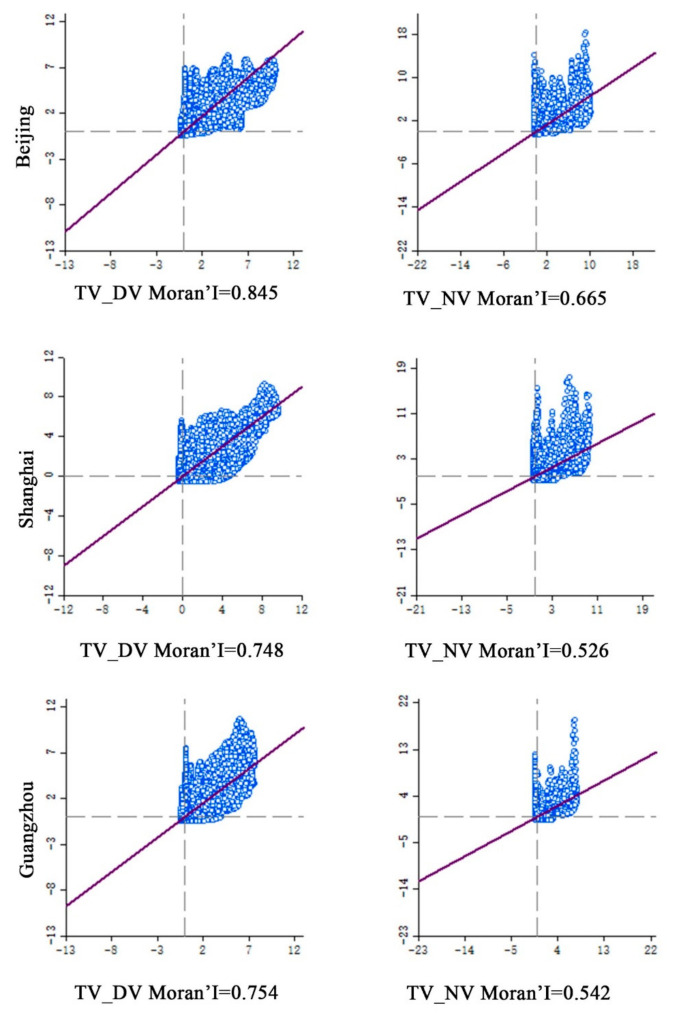
The global spatial autocorrelation between the urban takeaway vitality and urban vitality, with a significance of 0.05.

Figure 7 shows the scatter diagram of the Global Moran’s I between the takeaway vitality and urban vitality. The Moran’s I value is greater than 0 and the P value is less than 0.01, indicating that there is a significant spatial autocorrelation between the urban takeaway vitality and urban vitality. The distribution law of the high and low values of the urban takeaway vitality is consistent with the distribution law of the high and low values of the urban vitality, where high density is accompanied by high vitality. Therefore, the urban takeaway vitality and urban vitality have similar distribution law.

Based on the scatter diagram of Moran’s I index, although there is a significant spatial correlation between the takeaway activity and urban activity, there is a large difference in the spatial correlation degree between the urban activity and takeaway activity among Beijing, Shanghai, and Guangzhou (Figure 7). Among them, the TV-DV and TV-NV in Beijing is 0.845 and 0.665, respectively; the TV-DV and TV-NV in Shanghai is 0.754 and 0.542, respectively; and the TV-DV and TV-NV in Guangzhou is 0.748 and 0.526, respectively. We note that Beijing’s takeaway vitality has the highest correlation with the urban vitality while Guangzhou has the lowest correlation. Moreover, the correlation difference of vitality in different time periods is also obvious, which shows that the correlation between urban takeaway vitality and DV is much greater than that between urban takeaway vitality and NV. This may result from the difference in human activities between the day and night, as well as the closure of most takeaways at night and fewer takeout orders.

**Figure 8:**
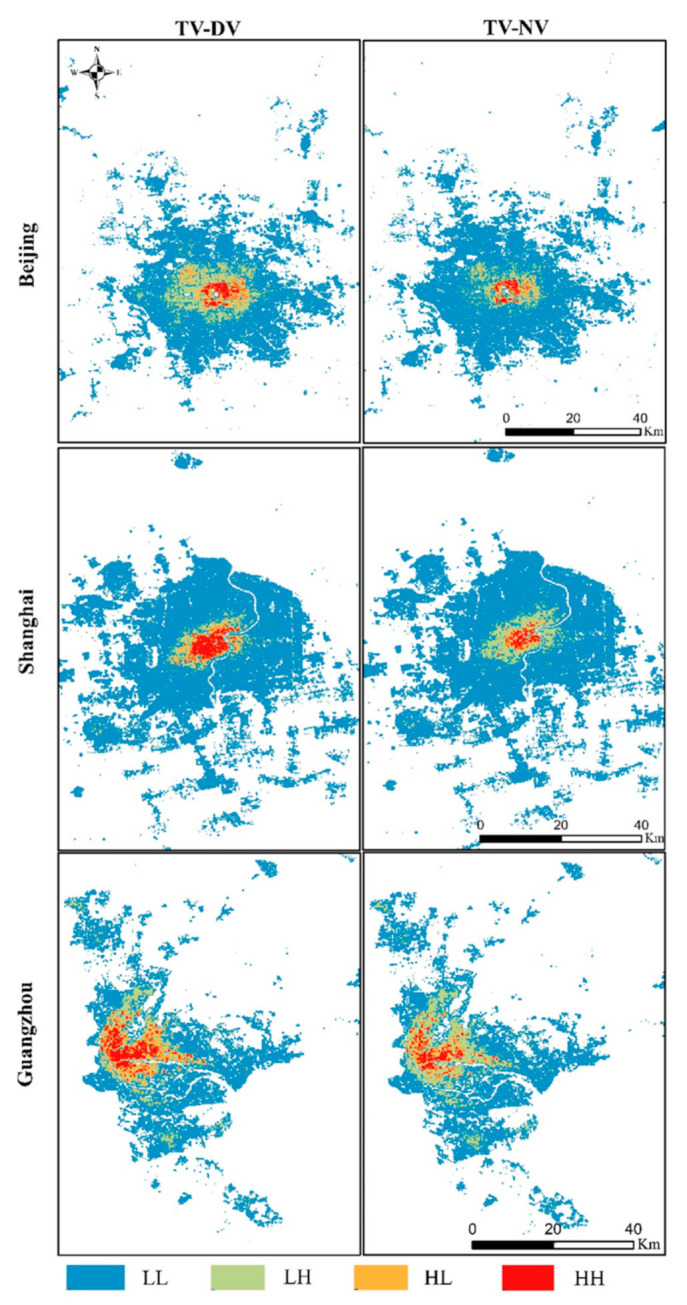
LISA mapping of the local spatial correlation between the urban takeaway vitality and the urban vitality.

Figure 8 shows the mapping of the bivariate binary LISA clustering relationship between the urban takeaway vitality and urban vitality. According to the LISA cluster mapping, the five categories of H-H, H-L, L-H, L-L, and insignificant were clustered and analysed. The following conclusions were obtained from the cluster mapping: (1) there are notable spatial similarities between the urban takeaway activity and the urban vitality in Beijing, Shanghai, and Guangzhou; (2) H-H hot spots are mainly concentrated in the urban centre and surrounding areas, which are significantly affected by the urban centre, while L-L cold spots are mainly concentrated in urban fringe areas; and (3) there is a large area of insignificant value cluster distributions between the urban centre and urban fringe in Beijing, Shanghai, and Guangzhou, which is similar in all three cities. In addition to the overall spatial autocorrelation and local correlation between H-H and L-L, the irrelevant distributions (H-L and L-H) between the urban takeaway vitality and urban vitality also appear in the cluster diagram. In Beijing, the distribution is mainly the L-H type while in Shanghai and Guangzhou, the distribution is mainly the H-L type during the day and the L-H type at night.

By comparing the clustering maps of Beijing, Shanghai, and Guangzhou, the clustering relationship varies in areas with different building and road densities. In areas with higher building and road densities, the clustering distribution can be characterised as H-H. In areas with lower building and road densities, the clustering distribution is L-L. In areas with a higher building density and a lower road density, the cluster distribution is mainly the L-H type. In areas with a lower building density and a higher road density, the clustering distribution can be mainly characterised as H-L. The reason for this is that the H-H cluster distribution is mostly located in the main urban area, the main urban area of a city is an area where urban functions and activities are concentrated. Therefore, the urban vitality and takeaway vitality of these areas have relatively higher values. The L-L cluster distribution is usually located in new urban areas and fringe areas. New areas of urban expansion often tend to be substantially larger than older urban areas, which produces less dense buildings and roads, thus restricting both the urban vitality and takeaway vitality. The L-H cluster distribution is mostly located in older urban areas, such as Hutong in Beijing. Although the building density is higher in these areas, there are few roads that can be used for delivery, resulting in a higher urban vitality value, but a lower takeaway vitality value. The H-L cluster distribution is mostly located in urban suburbs and new urban areas. Although the road density in these areas is higher, the building and residential densities are relatively low, which renders the urban vitality irrelevant to the takeaway vitality.

## 4. Discussion

As a special consumption behavior, urban takeaway has not only changed people’s lifestyle, but it also plays an important role in the healthy development of urban city as an integral part of urban space elements [53]. In this study, we obtained the degree of spatial correlation between the takeaway vitality and urban vitality by referring to the Meituan APP takeaway delivery method, which accounts for the delivery time and takeaway distance. Furthermore, we quantitatively analysed the areas accessible to takeaway to obtain the urban takeaway vitality value. Characterising the degree of spatial correlation cannot be performed until the urban takeaway vitality value is compared with the urban vitality. Finally, a new index reflecting urban vitality and urban spatial quality is summarized.

Although previous studies considered factors, such as the land use, mixing degree, density, block size, and building distribution, when calculating the urban vitality [73,74], these urban elements do not appropriately distinguish the activities of people in cities. With the wide application of mobile signalling, POI, population mobility, and other data, research on urban vitality tends to be based on different periods of crowd activity [23,35]. Through the spatial correlation analysis of the new takeaway vitality and urban vitality, we found that compared with the NV, the takeaway vitality is more closely related spatially to the DV, which shows that the takeaway vitality is more suitable for expressing a city’s DV.

Subordinate to the small-scale catering industry, although the urban takeaway vitality cannot reflect all aspects of a city, it still is an unattainable part of the urban catering industry, which is reflected in the face that it can be used as one of the important indicators to measure the vitality of a city [22]. Although there are certain restrictions on the people who choose the consumption behaviour of urban takeaway service, this special form of catering consumption can profoundly influence the life consumption behaviour of urban population, mainly reflected in that the development of the small-scale catering industry such as urban takeaway businesses and the development of urban areas are interrelated. First, the existence of urban small-scale catering industry will inevitably lead to substantial population flow activities and intensive urban activities [75]. Second, areas suitable for urban small-scale catering industry development will often promote other activities, such as recreation and rest [76]. Finally, compared with the large-scale catering and service industries, urban small-scale catering industry has a greater spatial flexibility [77,78]. With this kind of space flexibility, the transportation of catering industry can be more convenient, and the existing vitality of the city can be more accurately expressed, which is the direct embodiment of urban spatial quality. However, there is no doubt that not all urban populations have consumption behaviors, especially in countries and regions with a small population, that’s to say that urban vitality can’t be reflected only by urban catering industry. Urban vitality is the embodiment of urban macro spatial quality, and urban takeaway is only a part of it [79].

The value of this study is embodied in the following points: first, different from previous economics-based takeaway research perspectives, this study, for the first time, explores urban takeaway from urban planning and geography perspectives; second, compared with previous qualitative analyses of urban takeaway, this study proposes a quantitative analysis method for urban takeaway to calculate the vitality of urban takeaway. Finally, in terms of urban vitality, we propose a new index, i.e., the takeaway vitality, to express the urban vitality. The new index can provide important reference basis for the urban population and the rapid development of urban spatial quality. This study also provides an important research perspective for the related research on urban vitality, that is, urban vitality is not only reflected in urban functions and forms, but the consumption form, consumption capacity and consumption behavior of the city are all important manifestations of urban vitality, which will provide important reference value for the follow-up research on urban vitality [16,35].

Certain deficiencies in this study remain, which require continuous improvement. Urban vitality is a complex and abstract concept that encompasses multiple levels of society, economy and culture. The use of population mobility and night-time light can represent a portion of the urban vitality, but these are not sufficiently broad in their scope. Although factors, such as the diversity and liveability of the living environment, are also important aspects of urban vitality and urban spatial quality [65,80,81], owing to data availability, these two aspects were not been fully investigated, such that there may be some deviations in this study. In addition, it is concluded after data screening, that not all urban population have takeaway consumption behaviour. This kind of urban consumption with typical characteristics of Internet economy is more concentrated in the middle-aged and young people in the metropolis. and the takeaway data used in this study derives from the Meituan APP; however, a small number of merchants may be registered on other platforms, which may lead to incomplete data on takeaway in the target cities. Finally, although Beijing, Shanghai, and Guangzhou were selected for the case analysis in this study, future studies should select case points in central and western China for supplementary analysis to obtain findings that are relevant to more cities.

## 5. Conclusions

Multi-source data, such as Tencent-Yichuxing data, OSM data, building footprint data, urban takeaway distribution data, and night-time light data, were used in this study to quantitatively analyse the vitality of urban takeaway in China’s first-tier cities, i.e., Beijing, Shanghai, and Guangzhou, using the RRT algorithm, as well as a spatial correlation analysis with the urban vitality. Based on the results of this study, we can conclude the following:

(1) There is a significant spatial correlation between the urban takeaway vitality and urban vitality, but the correlation varies in different cities at different times;
(2) Different road densities and building densities also lead to different correlations.

Based on the use of multi-source big data, this study proposes a new urban vitality index based on the distribution of urban food takeaway delivery, i.e., the urban takeaway vitality. The quantification of the urban takeaway vitality and the analysis of its spatial correlation with the urban vitality conducted in this study further supplements relevant research on the urban vitality, which will be of significant value to the sustainable development of urban cities in the future.

## Figures and Tables

**Table 1:** Statistical descriptions of the data used in this study.

Data	Spatial Resolution	Data Source	Time
Takeaway	25 m × 25 m	https://www.meituan.com/	December 2020
Luojia-01	130 m × 130 m	http://59.175.109.173:8888/index.html	October 2018–October 2020
OSM	25 m × 25 m	https://www.openstreetmap.org/	2020
Building Footprint	25 m × 25 m	www.amap.com	2020
Tencent-Yichuxing	25 m × 25 m	Http://heat.qq.com/index.php	October 2020–December 2020

## Data Availability

Data Availability doi:10.5281/zenodo.4479726.
